# Astrocytic MicroRNAs and Transcription Factors in Alzheimer’s Disease and Therapeutic Interventions

**DOI:** 10.3390/cells11244111

**Published:** 2022-12-17

**Authors:** Ajmal Nassar, Triveni Kodi, Sairaj Satarker, Prasada Chowdari Gurram, Dinesh Upadhya, Fayaz SM, Jayesh Mudgal, Madhavan Nampoothiri

**Affiliations:** 1 Department of Pharmacology, Manipal College of Pharmaceutical Sciences, Manipal Academy of Higher Education, Manipal 576104, Karnataka, India; 2 Centre for Molecular Neurosciences, Kasturba Medical College, Manipal Academy of Higher Education, Manipal 576104, Karnataka, India; 3 Department of Biotechnology, Manipal Institute of Technology, Manipal Academy of Higher Education, Manipal 576104, Karnataka, India

**Keywords:** Astrocyte, Glutamate transporter 1, neurodegenerative diseases, neuroinflammation, microRNAs, Transcriptional Factors, long non-coding RNA, APOE4 nanodiscs

## Abstract

Astrocytes are important for maintaining cholesterol metabolism, glutamate uptake, and neurotransmission. Indeed, inflammatory processes and neurodegeneration contribute to the altered morphology, gene expression, and function of astrocytes. Astrocytes, in collaboration with numerous microRNAs, regulate brain cholesterol levels as well as glutamatergic and inflammatory signaling, all of which contribute to general brain homeostasis. Neural electrical activity, synaptic plasticity processes, learning, and memory are dependent on the astrocyte–neuron crosstalk. Here, we review the involvement of astrocytic microRNAs that potentially regulate cholesterol metabolism, glutamate uptake, and inflammation in Alzheimer’s disease (AD). The interaction between astrocytic microRNAs and long non-coding RNA and transcription factors specific to astrocytes also contributes to the pathogenesis of AD. Thus, astrocytic microRNAs arise as a promising target, as AD conditions are a worldwide public health problem. This review examines novel therapeutic strategies to target astrocyte dysfunction in AD, such as lipid nanodiscs, engineered G protein-coupled receptors, extracellular vesicles, and nanoparticles.

## 1. Introduction

Astrocytes, the largest and most numerous of the neuroglia, play a major role in regulating homeostasis and reacting to illness in the brain. microRNAs (miRs) are short nucleotide strands that control protein expression in a post-transcriptional manner and have an influence on astrocytes. Many miRs are involved in various neurological diseases [1,2]. Parkinson’s disease (PD) and Alzheimer’s disease (AD) are two comorbid conditions that are specifically correlated with the complex interactions of miRs in astrocytes [3]. Non-coding miRs, which are crucial participants in AD, influence the translation of two essential proteins implicated in Aβ-biogenesis, such as the APP-site cleaving enzyme (BACE1) and the Aβ-precursor protein (APP) [4,5,6]. miRs often inhibit the production of proteins by interacting with locations in the 3′-untranslated regions (3′-UTR) of mRNAs. According to reports, several miRs are under the regulatory control of astrocytes [3]. miRs have a cell-type-specific impact on proteins. miRs specifically present in astrocytes are responsible for AD gene modulations through diverse mechanisms. For instance, miR-298 therapy lowered BACE1 and native APP protein levels in astrocytes but not in neurons [7]. miR-181, miR-155, and many other miRs have recently been shown to have a role in the neuroinflammation processes in astrocytes during AD [8,9].

Neurons are fundamentally reliant on astrocytes for the supply of cholesterol since they are the main source of cholesterol constructors in the brain [10]. Astrocytic ApoE and brain cognition are closely related. Three main apoE isoforms (APOE2, APOE3, and APOE4) in humans have a significant impact on cognitive functions [11]. One of the AD pathogenic isoforms, apolipoprotein E4 (APOE4), enhances endosomal malfunction and impairs Aβ endocytic clearance [12]. The down-regulation of the Na + /H + exchanger (NHE6) and low-density lipoprotein receptor-related protein (LRP1) malfunction is responsible for the chronic acidification of endosomal pH in APOE4 astrocytes [12]. Astrocyte-derived cholesterol is necessary for the proper functioning of neuronal synapses because its absence may result in diminished synaptic activities [13,14,15]. In this review, we focused on the crucial miRs and how they contribute to the advancement of astrocyte-centered AD.

Apart from miRs, many transcription factors (TFs) have a vital function in astrocytes during AD. TFs are proteins that specifically anchor to DNA and influence gene transcription by attaching to the promoter region of genes. These perform an important function in directly controlling the degree to which genes are expressed [16]. TFs have been broadly implicated in development [17] and immunity processes [18]. It was shown that several transcription factors are selectively and highly expressed by astrocytes [19]. Therefore, analyzing the expression and regulation of TFs might be a therapeutic option for reducing astrocyte-mediated neurodegenerative disorders such as AD.

In the central nervous system (CNS), the main excitatory neurotransmitter glutamate starts fast signal transmission at synapses before the re-uptake by the glia in the area, notably astrocytes [20]. Neuroinflammation affects the CNS’s regular operation and is a crucial factor in the development of AD [21]. The primary CNS cells involved in inflammation and immunological responses are microglia and astrocytes [22]. Aβ stimulates a variety of astrocyte cell receptors, primarily, the receptor for advanced glycation end products (RAGE), which in turn triggers the stimulation of inflammatory factor nuclear factor-κB (NF-κB). This causes the astrocytes to release a broad range of pro-inflammatory cytokines and chemokines [23].

In this review, we highlight the critical role played by miRs in astrocytes in the primary APOE pathway, glutamatergic transport, and astrocytic or neuroinflammation processes that cause AD. We have also sought to evaluate the role of TFs that are unique to astrocytes involved in the pathogenesis of AD. Moreover, to open vistas in therapeutics, we come up with glimpses of drug-delivery systems to modulate the astrocytic miRs in AD.

## 2. The Role of Astrocytic microRNAs in Multiple Pathways in AD

### 2.1. Astrocytic microRNAs Mediate Cholesterol Metabolism in Alzheimer’s Disease

Astrocytes are the major producers of ApoE, which is principally linked to cholesterol-rich high-density lipoprotein (HDL)-like complexes and also the prime source of cholesterol transport in neurons [24]. Recently, a few studies demonstrated that cholesterol exposure caused astrocyte reactivity, boosted APP levels, and facilitated APP–BACE-1 interaction [25]. In a mouse model of AD, selective ablation of cholesterol production in astrocytes reduced the amyloid and tau burden. In astrocytes, the administration of cholesterol-free ApoE or the repression of cholesterol synthesis diminished the levels of cholesterol in cultured neurons and drove APP out of lipid clumps, where it bound with α-secretase and generated soluble APP-α (sAPP-α), an APP product that is a beneficial product for neurons [26]. A recent study also emphasized the significance of disruption in cholesterol metabolism in AD [27] and aged astrocytes [28]. AD is caused by both excess and a dysfunctional cholesterol pathway. Thus, it is important to maintain the cholesterol level in the brain [29].

Astrocytes release ApoE, which is then taken up by neurons. Astrocytes cause the trafficking of a broad range of miRs, and this astrocyte-specific miRs have a unique capacity to influence the genes in neurons [30]. Recently, astrocyte-released miRs, such as miR-126, was shown to suppress the genes involved in neuronal cholesterol production and promote the buildup of nuclear acetyl-coenzyme A (Acetyl-CoA) in neurons. This facilitates neuronal transcription and histone acetylation. Additionally, in mice, neuronal histone acetylation by astrocytic ApoE led to histone H3 protein subunit (H3K27ac) improvement at the promoters of numerous neuronal immediate-early genes (IEG) in neurons, which is likely associated with increased memory consolidation [31]. Intriguingly, ApoE4 in humans, one of the pathogenic isoforms of APOE transfers miRs from astrocytes to neurons at a rate lower than ApoE3, making it less effective at regulating metabolic and epigenetic processes in neurons [31]. APOE4 decreased miR-126 trafficking from astrocytes to neurons, which in turn reduced the histone acetylation at the promoter of several IEGs. Ultimately it led to decreased memory consolidation [31,32].

Aging has been attributed to the significant disturbance in the synthesis of cholesterol in the brain [27,28]. Age-related neurodegenerative issues have been linked to miR-335-3p, a neuron-enriched miR. Nevertheless, in cultured, aged astrocytes (cultured up to 35 days), increments in miR-335-3p are seen, which contributed to the decreased expression of the crucial cholesterol synthesis enzymes 3-hydroxy-3-methylglutaryl-CoA reductase (HMGCR) and 3-hydroxy-3-methylglutaryl-CoA synthase-1 (HMGCS1) and the reduced cellular cholesterol level. The conversion of acetyl-CoA to HMG-CoA by HMGCS1 and HMG-CoA to mevalonic acid by HMGCR is a critical step in the synthesis of cholesterol. Intriguingly, both HMGCS1 and HMGCR are inhibited by miR-335-3p. Increased miR-335-3p alters brain synapse functioning by repressing postsynaptic density protein (PSD-95) [10]. In the aging hippocampus, reduced miR-335-3p improves learning and memory [10]. In astrocytes, the significance of critical miR-335-3p in the metabolism of cholesterol and the concomitant alteration of cognitive capacities with aging give compelling evidence that epigenetic programming via miRs is essential for healthy cognitive functioning during aging [10].

For the synthesis of cholesterol in adult neurons, oligodendrocytes and astrocytes are essential [33], particularly astrocytes, which express apoE and can import cholesterol via receptor-mediated endocytosis of lipoproteins. After the apoE-cholesterol complex fuses with the lysosome, it is processed to release free cholesterol [34,35,36]. Lysosomal dysfunction causes significant cholesterol metabolism impairment [37]. The translation of neuronal miR-195 is most likely influenced by interactions between astrocyte-derived apoE and apoE receptors on neurons, and astrocyte-derived apoE4 exhibits a substantial decrease in neuronal miR-195 [38]. Lysosome size is strongly impacted by miR-195. The levels of miR-195 in cultivated iPSC-derived astrocytes of APOE4-expressing AD patients are lower. miR-195 is drastically decreased in neurons by astrocyte-derived APOE4, and associated lysosomal dysfunction is also seen in neurons. The development of lysosomal abnormalities in astrocytes as a result of APOE4-induced miR-195 decrease is startling. Both of these elements considerably contribute to AD [38].

**Figure 1:**
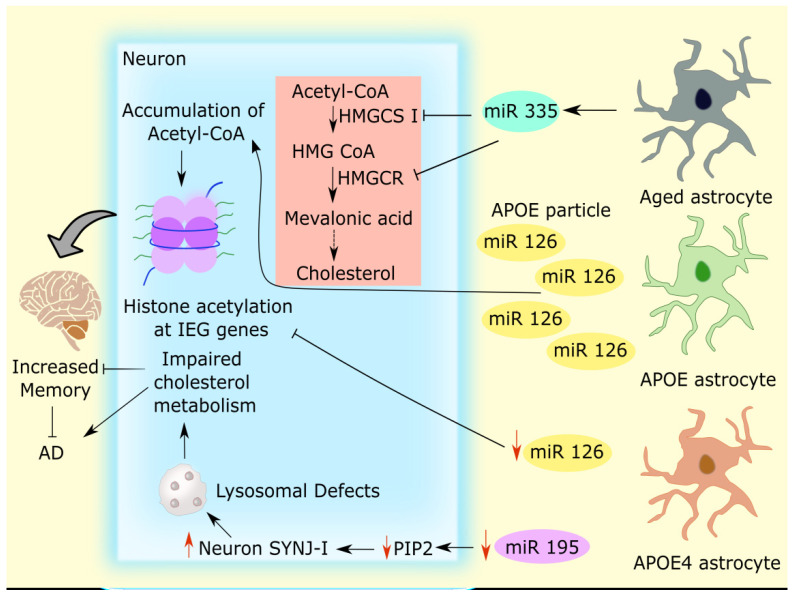
Astrocyte-neuron crosstalk through miRs and APOE-mediated cholesterol regulation. Astrocytes communicate with nearby neurons by delivering miRs. The APOE-induced astrocyte (GREEN) releases miRs, and miR-126 suppresses the genes involved in the generation of neuronal cholesterol, which in turn causes acetyl-CoA accumulation in neurons. As a result, increased H3K27ac enrichment and histone acetylation at several IEG genes (VIOLET) cause enhanced memory consolidation. However, APOE4-induced astrocytes (PALE RED) cause miR-126 trafficking from astrocytes to neurons to considerably decrease, which in turn reduces the histone acetylation at the promoters of IEGs. Ultimately, it leads to decreased memory consolidation. Aged astrocytes (PALE BLACK) display greater miR-335-3p levels in astrocytes. However, miR-335-3p hampers both HMGCS1 and HMGCR, which results in reduced cholesterol production leading to AD. Additionally, neuronal miR-195 is markedly reduced by astrocyte-induced APOE4, which caused PIP2 levels to drop and SYNJ1 levels to rise. Higher levels of SYNJ1 are associated with enlarged endosomes and lysosomal defects, which impair the metabolism of cholesterol in neurons and lead to AD. This picture shows how dysregulation of cholesterol production in astrocytes affects AD modulation in both protective and pathogenic aspects. miRs: microRNAs; AD: Alzheimer’s disease; ApoE4: ApolipoproteinE4; Acetyl-CoA: Acetyl-coenzyme A; H3K27ac: Acetylation of lysine 27 on histone H3 protein subunit; IEG: Immediate early gene; HMGCS1: 3-hydroxy-3-methylglutaryl-CoA synthase-1; HMGCR: 3-hydroxy-3-methylglutaryl-CoA reductase; PIP2: Phosphatidylinositol 4,5-bisphosphate; SYNJ1: Synaptojanin 1.

Additionally, lysosomal Phosphatidylinositol 4,5-bisphosphate (PIP2) regulates biological processes such as signal transduction, membrane trafficking, transporter activities, and ion channels in cell membranes [39,40]. ApoE4-induced specific alterations in the brain PIP2 homeostasis promote AD susceptibility [41]. In ApoE4 astrocytes, there was a notable reduction in miR-195, which led to a fall in PIP2 levels and an increase in Synaptojanin 1(SYNJ1), an enzyme that breaks down PIP2 in the brain. Surprisingly, increased endosome enlargement and ApoE4-linked cognitive impairments in AD are correlated with higher levels of SYNJ1 [42], and ApoE4-associated cognitive deficits in AD. In comparison to APOE4, APOE3 can significantly attenuate APOE4-induced SYNJ1 rise by increasing PIP2 [41]. The reduced levels of SYNJ1 could be beneficial in AD, especially in speeding the clearance of Aβ from astrocytes via the lysosomal degradation pathway [43]. All this evidence together conveys a clue that a decline in neuronal miR-195 by astrocytic APOE4 levels contributes to synaptic and cognitive impairment. Additionally, a decrease in astrocytic miR-195 levels leads to the accumulation of tau [38]. Interestingly, a recent article made the additional claim that a high-cholesterol diet causes a substantial decline in PIP2 levels [44]. A significant impact on cholesterol homeostasis in neurons may result from lysosomal disruption caused by the miR-195/PIP2-SYNJ1 pathway through APOE4 [38]. This identified an epigenetic cause of lysosomal dysfunction and the resulting disruption of cholesterol homeostasis in neurons. The interaction between neurons and astrocytes through miRs and APOE regulating cholesterol levels is depicted in Figure 1.

### 2.2. Astrocytic microRNAs in Dysregulated Glutamate Uptake in Alzheimer’s Disease

Astrocytes control glutamate release, reuptake, and recycling at tripartite synapses [45]. In rodents, significant glutamate transporters are expressed by presynaptic astrocytes, particularly excitatory amino acid transporter 2 (EAAT2), also known as GLT-1, in rodents [46] to modulate the levels of extracellular glutamate and synaptic activation. A glutamate surplus can result in the loss of neurons and synapses [47]. GLT-1 modulates the contacts of a normal synaptic network and guards against neurotoxicity by quickly removing extracellular glutamate. In AD, there is a considerable reduction in GLT-1, the main glutamate transporter in the cerebral cortex and hippocampus [48].

Three probable precursor hairpin sequences make up the miR-124 gene family: miR-124-1 (MI0000716) on chromosome 14, miR-124-2 (MI0000717) on chromosome 3, and miR-124-3 (formerly known as miR-124a, here MI0000150) on chromosome 2. MiR-124a was the previous name for miR-124-3p. The addition of the letter "a" or "b" to the microRNA identification number, as in the case of miR-124a and miR-124b, denotes the relationship and striking similarity of the mature sequences of the two miRs. The mature miR produced from the 5’ or 3’ arm of the hairpin loop is denoted by the letters 5p or 3p after the microRNA identification number, as in miR-124 -5p or miR-124 -3p [49,50,51,52,53].

**Figure 2:**
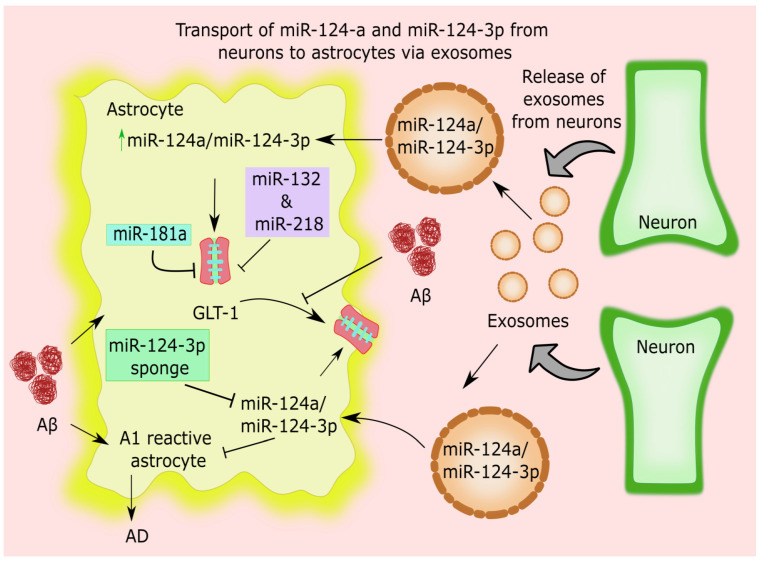
Astrocytic microRNAs in glutamate uptake in Alzheimer’s disease.

The preponderance of A1 astrocyte hyperactivity is a key factor in the pathogenic effects of AD [54]. miR-124-3p was discovered to be the highest enriched miR in neuron-derived EVs, and it plays a crucial function in blocking the in vivo activation of A1 astrocytes [55]. Astrocytes’ physiological activation of GLT-1 by neural impulses is very reliant. Aβ notably decreased GLT-1 expression in the astrocyte plasma membrane [56,57]. Aβ-42-mediated activation of GLT-1 mislocalization and internalization in astrocytes greatly reduces the rate at which they clear synaptically produced glutamate from the extracellular environment [58] (Figure 2).

miR-124-3p is neuron-specific, which is internalized into astrocytes, and up-regulates the glutamate transporter GLT-1 by repressing GLT-1 inhibiting miRs such as miR-132 and miR-218 derived from EVs [59,60]. Complementary patterns of miR-124-3p are generated to specifically block miR-124-3p exclusively in astrocytes using a sponge (SP) method. This further impedes GLT-1, resulting in neuronal damage [59,60]. In addition, miR-124-3p transfer, via EVs from neurons, plays a significant part in regulating neurodegeneration. These EVs can enter astrocytes directly, where they raise the levels of the proteins GLT1 and miR-124-3p [61]. In cultured astrocytes, direct miR-124-3p transfection also significantly and specifically raises GLT1 protein expression, but not mRNA, levels [61].

The transport of miRs from astrocytes to neurons was facilitated by EVs produced from astrocytes [62,63], but the manner and significance of the transfer of miRs from neurons to astrocytes is a source of astounding intrigue. GLT-1 is regulated by miR-181a in astrocytes, and an increase in mature miR-181a may be a factor in AD. In AD models, several synaptic proteins essential in synaptic plasticity and cognition are considerably downregulated by an age-dependent drop in GLT-1 and an increase in miR-181 [63,64]. Other neuron-specific miRs, such as miR-466, miR-669, miR-3082-5p, and miR-297a-5p, all of which boost GLT-1, were discovered in astrocyte-adopted EVs [60] (Figure 2).

This information offers a wealth of hints for astrocyte miRs, which are crucial for establishing a robust epigenetic environment in astrocyte essential genes that regulate numerous brain functions.

Aβ markedly decreases GLT-1 expression in the astrocytic plasma membrane by internalizing and mislocalizing GLT-1. miR-181a, miR-132, and miR-218 suppress GLT-1 in astrocytes, which are more prevalent in AD. miR-124a or miR-124-3p are liberated from neuronal EVs and penetrate astrocytes, where they boost GLT1 expression. miR-124-3p brings down GLT1-inhibiting miRs such as miR-132 and miR-218 (not shown) released from neuronal EVs, preventing the activation of A1 astrocytes. By suppressing miR-124-3p solely in astrocytes, a sponge containing complementary miR-124-3p sequences blocks GLT-1 and causes neuronal damage. GLT1: astrocyte-specific glutamate transporter 1; miR: microRNA; EVs: extracellular vesicles.

### 2.3. Astrocytic microRNAs Modulating Inflammation in Alzheimer’s Disease

AD is specified by the accumulation of Aβ and neurofibrillary tangles [64]. In astrocytes, the miR-181 family typically controls neuroinflammatory signaling, and it has been observed that the AD environment increases its expression [65]. Other than miR-181, numerous miRs, which are involved in AD pathology, including miR-146a [66], miR-146b [67], and miR-155 [68], were elevated in cortical astrocytes of mice following LPS treatment [69]. The frontal cortex–hippocampal circuitry is involved in learning and memory. The neurotransmitters from the cortex interact with the hippocampus. Several interconnecting pathways such as the glutamatergic, cholinergic, norepinephrinergic, GABAergic, and dopaminergic pathways interact with the frontal cortex–hippocampal circuit [70,71,72]. In AD-related neuroinflammatory events, miR-155 is one of the most researched immune-related miRs [73]. It was noted that the rise in miR-155 was peculiarly localized to the cortex and hippocampus of astrocytes [74,75].

The suppressor of cytokine signaling 1(SOCS-1) protein negatively controls the inflammatory gene response [76]. miR-155 was overexpressed and SOCS-1 was reduced when Aβ was administered to astrocyte primary cultures. In astrocytes treated with Aβ, silencing miR-155 results in a decrease in SOCS-1 level and a consequent decrease in proinflammatory cytokines [73]. Interestingly, the interconnection of miR-155 with the c-Jun terminal transcription factor was highlighted when astrocytes are activated by Aβ, along with the liberation of inflammatory mediators, such as IFN-β and IL-6. Levels of cytokines, including IL-6, correlated with neuroinflammation [77]. In Aβ-activated astrocytes, miR-155 levels and c-Jun levels were both markedly upregulated, which facilitated the synthesis of inflammatory mediators, including IL-6 and IFN- *γ*, as well as the development of extracellular Aβ -aggregates. c-Jun silencing in primary astrocyte cultures reduces the levels of miR-155 in Aβ-activated astrocytes [73]. Thus, targeting miR-155 can be a fascinating and an effective strategy for reducing AD-related neuroinflammation [73]. miR-146a’s role as an anti-inflammatory miR in a variety of neurological illnesses has been widely reported [78]. Nonetheless, miR-146a’s pro-inflammatory involvement in AD has recently been identified [79]. The choroid plexus (CP) is a critical region, which is a network of blood arteries, connective tissue, and cells found in the ventricles of the brain that is impacted by AD [80]. Exosomal miR-146a is reported to be secreted into the CSF by CP cells [79]. Astrocytes take up EVs produced by CP cells [81]. Environmental enrichment (EE) is a neuroprotective technique that has been successfully used to correct cognitive deficiencies in a variety of brain damage and illness models, including AD. Animals exposed to EE also engage in more physical activities and social interactions [82]. Increased miR-146a expression was seen in EVs generated from the CP after an EE. After being delivered to astrocytes, these EVs may prevent cognitive decline in AD model mice by decreasing astrocyte inflammation and boosting synaptic density in the subiculum area of the hippocampus. Additionally, they demonstrated that miR-146a therapy for Aβ/LPS-induced inflammatory astrocytes improved astrocyte inflammation by inhibiting NF-κB and tumor necrosis factor receptor-associated factor 6 (TRAF6), which restored synaptogenesis and reversed cognitive impairment [83]. In astrocytes, miR-146a targets the genes for interleukin-1 receptor-associated kinase 1 (IRAK1) and TRAF6 to reduce the expression of NF-κB [84]. As a causal pathology of inflammatory modulation, the activation of NF-κB leading to mobilization of NLRP3 inflammasome and subsequent release of IL-1β has been documented [85]. By boosting the expression of miR-146a and reducing the IRAK1, TRAF6, and NF-κB expression in astrocytes, EE might well be able to prevent cognitive damage [83]. Additionally, EVs enriched with miR-146a generated by bone marrow mesenchymal stem cells (BM-MSCs) in AD mice reduce astrocytic inflammation and enhance cognitive performance [86]. EVs released from BM-MSCs contain microRNA (miR)-146a, which is transferred into astroglia to reduce the inflammation brought on by diabetes [87]. BM-MSCs can be injected intracerebroventricularly to treat astrocytic inflammation because they adhere to the choroid plexus in the lateral ventricle and release EVs into the cerebrospinal fluid. Exosomal miR-146a released by BM-MSCs was taken up by astrocytes, and astrocytes were shown to have higher levels of miR-146a and lower levels of NF-κB. EVs from BM-MSCs in astrocytes can treat mitochondrial defects that cause diabetes-related astroglial damage [88].

It has been found that miR-592 significantly influences neurogenesis [89]. In astrocytes, the most important neuroinflammation and cell defense pathway is the KEAP1/NRF2 system, composed of the proteins kelch-like ECH-associated protein 1 (KEAP1) and nuclear factor erythroid 2-related factor 2(NRF2) [90]. Under oxidative stress, KEAP1 anchors NRF2 in the cytoplasm, to activate the antioxidant response element (ARE). This may boost the production of downstream antioxidative proteins such as heme oxygenase 1 (HO1) and NAD(P)H quinone oxidoreductase 1 (NQO1) [91]. The downregulation of the KEAP1 and concomitant NRF2 signaling activation regulates NF-κB transcriptional activity and reduces the activation of proinflammatory genes by cytokines [92,93,94,95]. Additionally, high levels of miR-592 and low levels of Dyslexia-Associated Protein KIAA0319 (KIAA0319) expression were found in the rat model of AD. In addition, miR-592 suppression increased N-NRF2 and NQO1 expression while decreasing C-KEAP1 expression, improving cell survival, and minimizing oxidative stress damage to astrocytes. In rat models of AD, downregulating miR-592 increased KIAA0319 through the stimulation of the KEAP1/NRF2/ARE pathways, which prevented damage of astrocytes induced by oxidative stress [96].

### 2.4. Astrocytic microRNAs- Long Non-coding RNA Interconnection in AD-linked Astrocytic Inflammation

**Figure 3:**
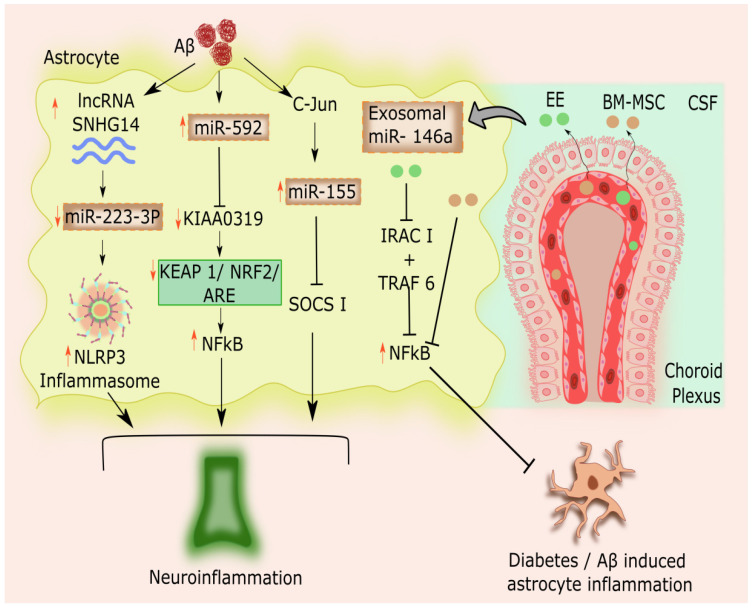
Astrocytic microRNAs-long non-coding RNA interconnection in astrocytic inflammation Alzheimer’s disease. EVs released from the CP into the CSF during EE and treatment with BM-MSCs reach astrocytes, where both EE and BM-MSCs protect against Aβ and diabetes-induced astrocyte inflammation through the elevation of miR-146a and concurrent reduction in NF-κB. EVs produced by EE mainly work by inhibiting IRAK1 and TRAF6, which lower NF-κB. In Aβ-activated astrocytes, levels of c-Jun and miR-155 were both noticeably increased, which in turn inhibited SOCS-1. miR-155 levels are decreased by c-Jun silencing, which also protects against neuroinflammation-induced AD. miR-592 expression was increased and KIAA0319 levels were lowered in AD rat models, which further activated KEAP1 and inhibited NRF2 signaling, leading to an increase in NF-κB expression. Additionally, miR-592 suppression activates KEAP1/NRF2/ARE pathways, which in turn reduces neuroinflammation and astrocyte damage brought on by oxidative stress. Finally, after exposure to Aβ, lncRNA, SNHG14 was elevated and miR-223-3p levels were lowered. Astrocytic miR-223-3p directly targets NLRP3 and reduces miR-223-3p expression further activating the NLRP3 inflammasome, which leads to neuroinflammation and AD. miR-223-3p levels were significantly elevated, and AD was prevented by SNHG14 knockdown. Aβ: Amyloid beta; SOCS-1: Suppressor of cytokine signaling 1; CP: Choroid plexus; CSF: Cerebrospinal fluid; EE: Environmental enrichment; NF-κB: Nuclear factor-κB ; TRAF6: Tumor necrosis factor receptor-associated factor 6; IRAK1: Interleukin-1 receptor-associated kinase 1; BM-MSCs: Bone marrow mesenchymal stem cells; KEAP1: kelch-like ECH-associated protein 1; NRF2: Nuclear factor erythroid 2-related factor 2; ARE: Antioxidant response element; KIAA0319: Dyslexia-Associated Protein; lncRNAs: Long non-coding RNA; MEG3: Maternally expressed gene 3; NLRP3: NLR family pyrin domain containing 3; SNHG14: Small nucleolar RNA host gene 14.

Long non-coding RNAs (lncRNAs) are a subclass of RNAs that are incapable of encoding proteins [97]. Recent research has shown that lncRNAs are crucial in controlling neuroinflammation and hence have a role in the etiology of many neurological conditions, such as ischemic stroke, cerebral hemorrhage, epilepsy, PD, and AD [98,99]. Maternally expressed gene 3 (MEG3), a lncRNA, is implicated and also seen to be reduced in a variety of cancer types. It also affects cell proliferation, progression, and prognosis [100]. MEG3 may act as a miR sponge, controlling the expression of the NLR family pyrin domain containing 3 (NLRP3) at the post-transcriptional stage [101]. NLRP3 and successive downstream cascades of inflammation take part in instigating the inflammation of AD [102]. MEG3 was shown to be downregulated in the hippocampal tissues of AD rats. The activation of astrocytes in the hippocampus of AD rats is inhibited by the upregulation of MEG3. By deactivating the PI3K/AKT signaling pathway and preventing astrocyte activation in the hippocampus, upregulating MEG3 in AD improves cognitive impairment. This suggests that the lncRNA MEG3 may be an effective AD marker and treatment target [103]. lncRNAs have been implicated in influencing inflammatory responses (Figure 3). Another lncRNA, small nucleolar RNA host gene 14 (SNHG14), mostly present in the cytoplasm of astrocytes, acts as a sponge for miR-223-3p. MiR-223-3p specifically targets NLRP3 in this circumstance (Figure 3). It was shown that SNHG14 was upregulated because of Aβ exposure. Overexpression of SNHG14 dramatically decreased miR-223-3p levels, whereas SNHG14 knockdown markedly increased miR-223-3p levels and prevent AD [104].

The only two identified unique astrocyte-linked lncRNAs are MEG3 and SNHG14. While lncRNAs that play a significant role in preventing AD were validated [105], they are not astrocyte-specific. Therefore, lncRNA, which is exclusive to astrocytes and their function in the modulation of AD, needs a lot of attention.

## 3. Astrocyte-Specific Critical Transcription Factors in Alzheimer’s Disease

### 3.1. STAT3

The abnormal activation of astrocytes in the AD brain is responsible for neuroinflammation and cognitive impairment. The suppression of the signal transducer and activator of transcription 3 (STAT3) enhanced cognitive impairment in APP/PS1 mice and reduced neuroinflammation [106]. Additionally, inhibition of Tyk2, which mediated STAT3 activation, attenuates Aβ-enhanced neuronal cell death [107]. The inhibition of phosphorylation of STAT3 at Ser 727 reduced the GFAP expression in the LPS-treated glial cell and APP/PS1 mouse model, facilitating the decrease in Aβ deposition and cognition impairment [108] Table 1. Interestingly, astrocyte differentiation promotes STAT3, which increases the gene expression of GFAP. The activation of the JAK/STAT pathway increases GFAP as well as reactive astrogliosis. Furthermore, an upregulation of the suppressor of cytokine signaling 3 (SOCS3) inhibits the JAK/STAT3 pathway primarily in astrocytes and reduces the STAT3 and GFAP levels in mouse models [109,110].

### 3.2. PAX6

Paired box 6 (PAX6) is a controlling factor for neurogenesis that inhibits proliferation and encourages the maturation of astrocytes in mice. Research observations show that knockdown conditioning of Pax6 decreased various markers involved in neurodegeneration such as S100 β, BDNF, p73δ, p73α, NGN2, and GFAP in astrocytes. This reflects the importance of Pax6 in ameliorating neurodegenerative diseases such as AD [111].

### 3.3. TFEB

Transcription factor EB (TFEB) regulates the autophagy and lysosomal pathways. It is known to be highly abundant in astrocytes and neurons. Notably, TFEB is implicated in the pathogenesis of AD via an amyloid pathway. The overexpression of TFEB in astrocytes has shown its beneficial effect in Alzheimer’s by clearance of Aβ via enhancing the lysosomal degradation in the mouse model [112,113].

### 3.4. TFAM

The overexpression of mitochondrial transcription factor A (TFAM) in astrocytes can protect mitochondria against Aβ1-42 peptide-mediated damage to neurons. In addition, TFAM diminishes the production of reactive oxygen species and promotes mitochondrial biogenesis [114].

### 3.5. NFIA

Astrocyte-specific nuclear factor I-A (NFIA) provides region-specific properties to hippocampal astrocytes important for memory and learning. Depletion of NFIA resulted in a reduction in memory and learning in hippocampus astrocytes [115].

### 3.6. CEBPβ

A study has found that elevated CCAAT-enhancer-binding protein (CEBPβ) expression promotes the interconnection of CEBPβ with the NF-κB p65, resulting in the upregulation of the transcription of proinflammatory cytokines secreted by the astrocytes. A nanocarrier-packaged carnosic acid alleviates microglia and astrocytes-associated neuronal degeneration and decreases Aβ deposition by suppressing the CEBPβ-NF-κB signaling and further improves cognition in AD [122]. Indeed, CEBPβ is associated with the pathogenesis of AD by enhancing the delta-secretase expression in the mouse model [116].

### 3.7. YY-1

A transcriptional factor Yin Yang 1 (YY-1) binds to the BACE1 promotor and enhances the BACE activity in astrocytes, resulting in the generation of Aβ peptides. Interestingly, reduced BACE1 expression enhances the cholinergic pathway and memory. Therefore, displacing the binding of YY-1 with BACE1 promotes a therapeutic benefit in treating AD [117,118]. 

## 4. Epigenetic Astrocyte Transcription Factors in Neurodegeneration

Epigenetic mechanisms are implicated in the maintenance of neuronal growth as well as memory encoding and cognition. However, the dysregulation of epigenetic mechanisms relates to neurodegenerative diseases. Blocking the epigenetic alterations can serve as a therapeutic target in attenuating neurodegenerative diseases [123]. Studies have reported that epigenetic processes influence astrocyte responses in neurodegenerative disorders. Epigenetic mechanisms may provide novel pathways to control astrocyte-associated neuroinflammation and neurotoxicity, combined with the epigenetic marks implicated in astrocyte formation [110]. The following are the epigenetic astrocyte transcription factors in neurodegeneration.

### 4.1. HDAC

Aβ promotes the HDAC-2 and HDAC-5 expression in the cell nucleus by binding to the booster region of BDNF and inducing H3 deacetylation, resulting in lower expression of BDNF in astrocytes. Research has shown that HDAC inhibitor (trichostatin) antagonizes Aβ promoted HDAC-2 and HDAC-5 expression-induced H3 deacetylation in astrocytes, and further increases the expression of BDNF [119].

### 4.2. CREB

The cAMP response element binding protein (CREB) is an important transcriptional factor in long-term memory, and it maintains the expression of growth factors such as BDNF, which is important for the maintenance and development of long-term memory and plasticity [124]. CREB signaling in astrocytes is a crucial transcription factor that mediates neuroprotection and synaptic plasticity. Inactivation of CREB transcription occurs by two mechanisms: First, Aβ induces the CREB-dependent transcription shortage and promotes AD. Moreover, the modulation of astrocytic CREB has shown a beneficial role. Second, by boosting the HDAC4 nuclear translocation inhibits the binding of CREB to its co-activator CBP, which is essential for its activation and remarkably associated with cognition impairment in the developing brain [120].

### 4.3. Nurr1

Tauopathy is mostly coupled to nuclear receptor-related 1 (Nurr1) protein dysregulation in AD patients. It is evident that Nurr1 is regulated by CREB-dependent pathways and controls memory and learning functions in astrocytes. Surprisingly, the inhibition of HDAC using trichostatin A increases Nurr1 protein and drives memory. Additionally, the inhibition of Nurr1 in astrocytes promotes pro-inflammatory cytokines, which are toxic to neurons and induce neuronal death [121].

All the Astrocyte-Specific Critical Transcription Factors in AD has been tabulated in Table 1.

## 5. Novel Therapeutic Strategies to Target Astrocyte Dysfunction in Alzheimer’s Disease

### 5.1. APOA1 Nanodiscs in Astrocytes

AD is most likely caused by astrogliosis, which is identified by a deficiency in cholesterol synthesis and its transient in the brain [125]. Apolipoprotein A-I (APOAI), one of the prominent protein components of HDL, has an integral role in AD [126]. The production of apoproteins and the assembly of lipoproteins are necessary for the transit of cholesterol in the brain parenchyma. Surprisingly, cerebral synthesis of cholesterol mainly occurs under the supervision of astrocytes [31,127,128]. 

APOA1 aids the trafficking of freshly synthesized cholesterol from the endoplasmic reticulum/Golgi apparatus (ER/Golgi) to the cytosol by building a linkage with ATP-binding cassette transporter A1 (ABCA1) on the cellular membranes of astrocytes [127]. In APP/PS1 rats with APOA1 loss, astrogliosis as well as amyloid-induced astrocyte reactivity is more prevalent in the cortical and hippocampus regions [126]. A lipid nanodisc or Nanolipoprotein particle (NLP) is a discoidal lipid bilayer that is supported at one edge by proteins, peptides, or polymers. An apolipoprotein-bound lipid bilayer is contained in a patch on the lipid nanodisc. Recent research has led to the development of both APOA1 and APOE4 NLPs [128]. Nanodiscs provide non-detergent membrane-imitating environments and have significantly improved the structural research on membrane proteins [129]. There are two significant links between structural biology and nanodiscs. The first is connected to HDL, a particle that serves several purposes, including transporting lipids, and nascent HDL, a nanodisc that is kept stable by APOA1 [129].

Numerous ATP-binding cassette transporters are needed for the development of discoidal lipoproteins. Astrocyte membrane has prominent ABCA1 transporters [130]. To construct discoidal lipoproteins, cholesterol must always be diverted from astrocytes to apolipoproteins via ABCA1 [131], which enables phospholipid and cholesterol incorporation into maturing discoidal lipoproteins. In addition, those with AD exhibited reduced amounts of lipoprotein [132]. Recently, cholesterol acceptors that mimic biomimetic HDLs (APOA1 nanodisc) were created. The incorporation of APOA1 nanodiscs into astrocytes appears to offset the downturn in cerebral HDLs. The efflux of cholesterol from astrocytes is augmented by APOA1 nanodiscs after they cross the blood–brain barrier, making them an appropriate perspective-supportive treatment for AD [133]. 

To re-establish cerebral cholesterol transport, APOA1 nanodiscs that imitate cerebral HDLs are used. APOA-I, an apoprotein with neuroprotective features such as preventing Aβ aggregation, is used to make lipidic nanodiscs [134]. After employing APOA1 nanodiscs, a substantial increase in the efflux of cholesterol from healthy human astrocytes was observed. Additionally, they were just as effective in increasing cholesterol efflux as astrocyte-conditioned media, indicating that their function is comparable to that of APOE-lipoproteins made by astrocytes [135]. It is interesting to note that APOA1 nanodiscs allowed cholesterol efflux from NHA cells following exposure to Aβ 1–42 oligomers. As the Aβ dose was increased, the effectiveness of APOA1 nanodiscs to enhance cholesterol efflux from Aβ-exposed cells was diminished. The ABCA1 transporter, which moves cholesterol from cells to growing lipoproteins, as well as the reduced numbers of the ABCA1 transporters itself, are what ultimately cause the faulty cholesterol ejection from reactive astrocytes [134,136,137]. The increased cholesterol efflux is explained by the treatment of nanodiscs, which boosted ABCA1 levels. In addition, a decline in GFAP levels was found [136]. The propensity of APOA1 nanodiscs to pass the BBB is still most apparently due to the operation of SR-B1, also referred to as the HDL scavenger receptor class B type 1 receptor, which predominantly helps HDL transcytosis at the BBB. By increasing astrocyte reactivity and lowering the concentrations of ABCA1 transporters, Aβ oligomers may have an impact on the efflux of cholesterol from astrocytes. These circumstances could account for the diminished expression of cerebral lipoproteins scrutinized in AD patients [135]. Additionally, brain apoprotein levels and lipidation status may influence how the disease develops [137]. The development of APOA1 nanodiscs, which can penetrate the BBB in vitro and imitate cerebral discoidal HDLs in form and function, maybe a potential method to offset AD’s altered cholesterol transport [136].

**Figure 4:**
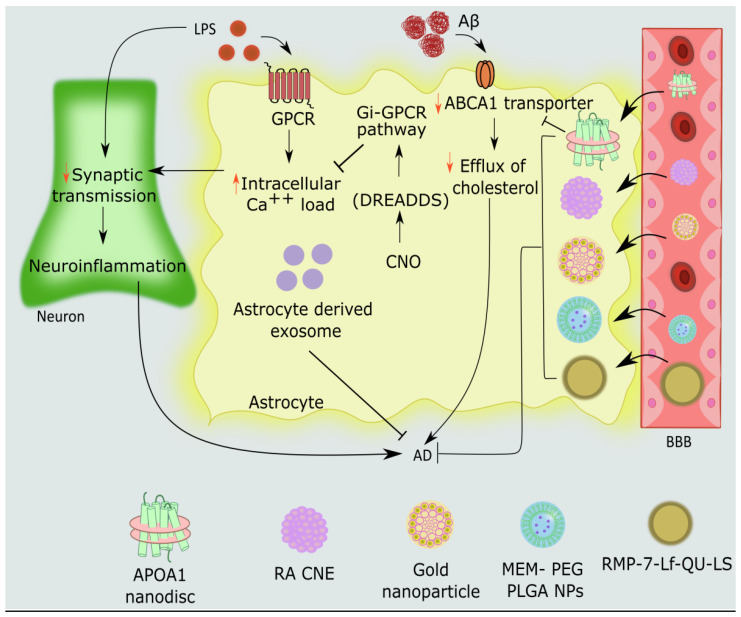
Novel therapeutic strategies to target astrocyte dysfunction in Alzheimer’s disease. Reactive astrocytes are brought on by the administration of Aβ 1–42, and as a result, the levels of the ABCA1 transporter are lowered, inhibiting the release of cholesterol from astrocytes, and hence impeding cholesterol export. When APOA1 nanodiscs cross the BBB, they improve ABCA1 transporters, preventing the reduction of cholesterol efflux in astrocytes caused by exposure to Aβ 1-42. LPS disrupts the Gi-GPCR pathway, which in turn causes astrocytes to have significantly higher intracellular calcium levels and the accompanying neuroinflammation. Chemogenetic stimulation of the astrocytic Gi pathway by Gi- DREADD agonist CNO reduces intracellular calcium load in hippocampal astrocytes and concomitant neuroinflammation, which enhances synaptic transmission and prevents AD. MEM-PEG-PLGA nanoparticles, glucose-coated gold nanoparticles, RMP-7-Lf-QU-LS, and RA CNE can all cross the BBB and enter astrocytes to prevent Aβ-induced toxicity in neurons and astrocytes.GPCRs: G protein-coupled receptors; APOA-I: Apolipoprotein A-I; ABCA1:ATP-binding cassette transporter A1; NLP: Nanolipoprotein particle; DREADDs: Designer receptors only activated by designer drugs; CNO: Clozapine N-oxide; Gi: Inhibitory G protein; MEM-PEG-PLGA-NPs: Memantine–polyethylene glycol–Polylactic-co-glycolic nanoparticles; RMP-7-Lf-QU-LS: Quercetin (QU)-encapsulated liposomes(LS) grafted with RMP-7 and lactoferrin (Lf); RA CNE: Chitosan-coated rosmarinic acid nanoemulsions.

In addition to lipid nanodiscs, formulations to combat the effects of AD on astrocytes have also been made using lipid nanotechnology. Targeting brain cells requires non-invasive, biocompatible therapeutic methods, and liposomes are well-suited because of their unique surface functionalization and sustained, selective delivery [138]. Suesca et al. reported that astrocytes have facilitated the uptake of liposome-containing drugs [139]. Quercetin (QU)-encapsulated liposomes (LS) containing RMP-7 (bradykinin analog) and lactoferrin (Lf) or (RMP-7-Lf-QU-LS) had the unique ability to cross the blood–brain barrier and enter a specific astrocytic location, where they could limit toxicity to human astrocytes and prevent AD [140]. Furthermore, using lipid nanotechnology, rosmarinic acid (RA) loaded nanoemulsions were produced. The polyphenolic molecule RA, which is present in nature, has been found to have anti-inflammatory and antioxidant characteristics that aid to protect the AD brain [141]. Chitosan-coated RA nanoemulsion (RA CNE), designed for nasal delivery as a novel neuroprotective therapy, proved successful in preventing LPS-induced astrocyte hyperstimulation and reinstating astrocyte redox state in primary astrocyte cultures [142]. Furthermore, LPS-induced memory loss, neuroinflammation, and oxidative stress were all mitigated when RA CNE is administered via the nasal route in Wistar rats [143]. Various delivery systems to target astrocytic dysfunction in AD are depicted in Figure 4.

### 5.2. Engineered G Protein-Coupled Receptors (GPCRs) in Astrocytes- Chemogenetic Approaches

Chemogenetics is a relatively recent technique that makes use of altered G-protein-coupled receptors (GPCRs) [144,145]. Sophisticated chemogenetics can be employed to modulate GPCR signaling with the help of DREADs, also termed “designer receptors only activated by designer drugs” [144,145]. The GPCR’s three primary signaling pathways (Gi, Gq, or Gs) [146] were modulated using DREADDs. A selective ligand of choice for DREADDs is clozapine N-oxide (CNO) [147]. To investigate how the brain works and behaves, this method has frequently been employed to control the activity of different types of neurons [148,149]. However, nowadays, it is a unique study paradigm to control astrocyte GPCR signaling [150,151]. A decrease in neuroinflammation was observed after GPCR modulation solely in astrocytes [152]. Neuroinflammation was effectively regulated via a Gi-coupled DREADD in astrocytes. In a mouse model of LPS-induced neuroinflammation, Gi- DREADD was expressed in hippocampus astrocytes. When astrocyte Gi-DREADD is stimulated by clozapine N-oxide (CNO), neuroinflammation is suppressed, which is supported by a drop in proinflammatory cytokine levels. Furthermore, this technology contributed to decreased glial activation, which led to the prevention of cognitive impairment in mice. Gi-DREADD activation in primary astrocytes markedly reduced LPS-triggered NOS2 activation and concurrent nitric oxide generation [150,151]. Because astrocytic Gi-GPCR activation has a substantial effect on regulating neuroinflammation, it is possible to identify new targets to modulate neuroinflammation [150,151]. Additionally, Gi-DREADD seriously influences astrocytes in the Schaffer collateral region. Gi-DREADD facilitates synaptogenesis and hippocampus plasticity and the concurrent establishment of contextual fear memory. Further, Gi-DREADD suppresses distant but not recent memory recall by modulating hippocampal CA1 astrocytes during learning and by lessening the activity of CA1 neurons projecting to the anterior cingulate cortex (ACC) during memory retrieval [153]. Modulation of the Gi signal by long-term activation of Gi-DREADD may restrict astrocyte activation and the ensuing neuroinflammatory abnormalities.

The activation of the astrocytic Gi-circuit using this sophisticated chemogenetic technique significantly reduced neuroinflammation, one of the primary effects of LPS-induced calcium overload [146]. However, Gq-DREADDs agonists, such as Gi-DREADDs, have a massive potential to consistently affect spontaneous astrocytic Ca^2+^ events, implying that astrocytes can respond with Ca^2+^ via both Gq- and Gi-coupled DREADDs [154,155,156,157]. Astrocytic Ca^2+^ transients may become more frequent when Gq-coupled GPCRs are activated [158]. It was surprising to learn that Gq- and Gi-DREADD stimulation of CA1 astrocytes strengthens the lengthy synaptic potentiation in the hippocampus CA1 Schaffer collateral circuit, which is independent of calcium-controlled neuroinflammation signaling. This suggests that the stimulation of the Gq or Gi pathways triggers synaptic potentiation in both Ca^2+^ -dependent and Ca^2+^ -independent manners [159].

The blood–brain barrier (BBB) is a multicellular vascular barrier that distinguishes the CNS from external blood circulation. By precisely supervising the flow of molecules and ions, instantaneously delivering oxygen and nutrients based on the requirements of the neurons, and safeguarding the brain from toxins and pathogens, the BBB maintains an environment that allows neurons to operate effectively [160]. The transport of therapeutic biomolecules and transgenes to the brain is currently a significant problem. The microvascular endothelium, pericytes, and astrocytes make up the BBB, which in turn blocks the passage of most hazardous substances and larger hydrophilic molecules [161]. Between 90 and 98% of the brain’s microvasculature and sheaths are made of astrocyte glial cells [138]. The cellular connections between blood arteries and neural networks are strengthened by astrocytes. Both enhancing astrocyte–endothelial cell interactions and decreasing trans-endothelial permeability are ways that pericytes affect the integrity of the BBB [162]. Additionally, brain endothelial cells have limited pinocytotic activity [163]. The endothelium barrier prevents the entry of numerous medications and larger macromolecules, such as cytokines and gene-modifying treatments, which have great potential for treating CNS diseases [164,165,166].

Mature astrocytes play a specialized function in preserving the integrity of the BBB through the up-regulation of proteins via various signaling pathways [167]. Para glycoprotein (Pgp) and GLUT1 are two transporters whose expression and polarized localization are influenced by astrocytes [168,169]. However, it is yet unclear if the decrease in GLUT1 and Pgp affects disease pathophysiology, particularly AD. AD is caused by the overexpression of the Aβ precursor protein in the absence of GLUT1 and Pgp, and desirable levels eliminate and clear the Aβ [170,171].

### 5.3. Extracellular Vesicles

EVs produced by astrocytes interact with neurons by encouraging the formation of ceramide and sphingosine in neurons, which benefits excitatory neurotransmission [172]. It has been proposed that the dysregulation of ceramide and sphingomyelin levels plays a role in the etiology of AD [173]. Current studies have found that in cell–cell signaling, EVs play a crucial role in disease progression. Moreover, in the therapeutics of various disorders, the role of EVs has been identified [174]. Astrocyte-derived EVs have both neuroprotective [175,176,177] and neurodegenerative effects [178]. When astrocyte EVs connect with neuronal and microglial EVs through capillaries, they can contribute to the inflammatory response [179]. Astrocytic EVs that detect and react to neuronal activity and take part in the reuptake of neurotransmitters can interact with neuronal EVs. According to Croft et al., TGF- β enhances Aβ clearance and decreases Aβ accumulation in AD circumstances [180]. The ability of astrocytic EVs to boost TGF- β signaling in neurons through the transportation of the synaptogenic carrier Fibulin-2 and subsequent acceleration of synaptogenesis was discovered recently [181]. Eminently, LPS and IL-6 activation of pro- and anti-inflammatory pathways, respectively, is the trigger for astrocyte EV release. Additional mechanistic investigations revealed that EVs produced by astrocytes transport bioactive chemicals to neurons, such as glutaminase, prostaglandin D2 synthase, and others, enhancing neural plasticity and neuronal functions [176,182]. Venturini et al. reported Neuroglobin, a protein acting as a neuroprotectant against cell injury, was found in the EVs produced by astrocytes. The prospect that EVs might transmit neuroglobin to neurons would add a mechanism to the putative astrocytic neuroprotectant activity [182], and diminish the Aβ neurotoxicity in vitro and in vivo [183]. EVs from cultured astrocytes have previously been demonstrated to interact with co-cultured neurons to encourage the growth of nearby neurons [184].

Heat shock proteins and other neuroprotective substances are known to be shuttled by astrocyte-derived EVs, which may mitigate the cytotoxicity of abnormal proteins and avert neurodegeneration. HSP70, synapsin-1, ApoD, and mitochondrial DNA are just a few of the proteins and RNAs that are found in astrocyte-derived EVs that shield neurons from cellular stress and degeneration [185]. HSP70 protects neurons from cellular stress and eventual death; improves the clearance of Aβ and monomeric, prefibrillar oligomeric tau inhibition and its aggregation; and plays cytoprotective roles in AD. Conversely, AD is accompanied by synaptic dysfunction caused by synapsin I cleavage fragments [62,186,187]. The EVs generated from astrocytes release apolipoprotein D (ApoD), which reduces oxidative stress, neuroinflammation, neurodegeneration, and a protective role in AD and increases with aging [188,189].

Prostaglandin E2/prostaglandin E2 receptor 4 (PGE2/EP4) signaling is a highly desired signaling pathway for preserving MSC homeostasis [190,191,192]. Unexpectedly, a striking discovery was explained in the context of AD by altering MSC homeostasis by suppressing EP4 signaling. After being given an EP4 antagonist, MSCs lost their stem cell characteristics. However, the release of MSC-EVs in response to EP4 signaling suppression also has important therapeutic ramifications. When given to animals with hippocampal injury, systemic treatment of EP4 antagonist-elicited MSC-EVs reinstate cognitive, learning, and memory deficits and bring down the reactive astrogliosis and concurrent inflammation by diminishing the production of reactive A1 astrocyte-inducers, GFAP, and vimentin in astrocytes [186]. Conversely, EVs containing the Prion protein, laminin receptor, and other neural protective proteins are released by astrocytes in H2O2-induced hypoxia and ischemia conditions to improve neuronal cell survival [187]. Prions are surface receptors of Aβ oligomers, and the laminin receptor helps with the internalization of Aβ receptor [193,194]. In diseased situations, astroglial EVs also cause severe dysfunction in cortical neurons in addition to their beneficial effects. EVs obtained from co-cultures of neurons with mostly astrocytes that were exposed to Aβ substantially worsened synaptic integrity in the AD model. In response to IL- 1β, astrocytes secreted EVs that prompted neurons to generate neurotoxic Aβ. This study provided a potential mechanism for the disturbance of neurotransmission brought on by astroglial EVs in AD-induced neuroinflammation [195].

### 5.4. Nanoparticles

Memantine has been indicated in AD but has not been proven to be 100 percent effective in treating moderate to severe AD. Lopez et al.’s investigation showed that Polylactic-co-glycolic (PLGA) nanoparticles can serve as an appropriate solution in astrocytic cell lines by increasing the drug’s effectiveness at the target location while reducing side effects. Due to these factors, memantine was loaded into biodegradable PLGA nanoparticles that were created using the double emulsion process and had polyethylene glycol (PEG) coated to the surface. When administered orally, MEM-PEG-PLGA nanoparticles (NPs) were designed to target the BBB for the treatment of astrocyte damage induced by AD [196].

The BBB has been the subject of extensive research, including the use of nanoparticles as transporters. Glucose-coated gold nanoparticles cross the human brain endothelium and enter the astrocytes [197]. Gold nanoparticles are thought to be viable options for treating neurodegenerative disorders. They have a synergistic effect on suppressing Aβ aggregation and dissociating Aβ fibrils, in addition to preventing deficits in recognition and spatial memory seen in astrocytes. They also have exceptional optical qualities, high chemical stability, excellent electrical conductivity, biocompatibility, and catalytic activity. They have a large surface area and a strong capacity for protein adsorption. To find Aβ proteins, they can be combined with antibodies. They also enhance signal and electron transfer effectiveness in astrocytes [197,198,199,200]. Muller et al. reported that in sporadic AD, gold nanoparticles protect against cognitive impairments, oxidative stress, and inflammation [201].

## 6. Conclusions

miRs have generated keen interest in the treatment of AD since they are abundantly expressed in neurons. However, recent research indicates that astrocytes, in addition to neurons, have a substantial responsibility for controlling AD. This review highlights how different miRs are delivered from astrocytes to neurons in regulating cholesterol production, glutamatergic transmission, and inflammatory signal transduction through astrocyte–neuron cross-talk mechanisms in AD. Furthermore, some crucial transcription factors have been discovered to play a vital role in astrocytes in tandem with AD through both non-epigenetic and epigenetic means. Recently, novel therapeutic techniques have been developed, including the chemogenetic activation of astrocyte signaling utilizing DREADD and lipid nanodiscs. Along with this, novel formulation techniques have been created to address the dysfunction of the glutamatergic system, the impairment of cholesterol metabolism, and the anti-inflammatory strategy to prevent astrocyte defects, which are contemporaneous with AD. These crucial techniques have recently provided invaluable findings and much optimism to explore the treatment choice for AD through astrocytic signaling manipulation. However, there is still a dearth of conclusive studies in this field; therefore, particular strategies must be utilized to entangle neuronal dysfunction.

## Figures and Tables

**Table 1:** Astrocyte-specific transcription factors in AD.

Sr.no	Astrocyte Transcription Factor	Role in Astrocytes	Reference
1	STAT3	Increases reactive astrocytes, Aβ deposition, and cognition impairment.	[106,107,108]
2	PAX6	Control neurogenesis and decrease neurodegenerative markers	[111]
3	TFEB	Clearance of Aβ plaques via lysosomal biogenesis.	[112,113]
4	TFAM	Protects mitochondria against Aβ-42 peptide via mitochondrial biogenesis	[114]
5	NFIA	Enhances learning and memory	[115]
6	CEBPβ	Upregulate pro-inflammatory cytokines	[116]
7	YY-1	Generation of Aβ peptides	[117,118]
8	Epigenetic HDAC	Inhibits Aβ induced HDAC-2 and HDAC-5 expression	[119]
9	CREB	Enhances long-term memory	[120]
10	Nurr1	Controls memory and learning functions	[121]

Abbreviations: STAT3: *S*ignal transducer and activator of transcription 3; PAX6: Paired box 6; TFEB: Transcription factor EB; TFAM: Mitochondrial transcription factor A; NFIA: Astrocyte-specific nuclear factor I-A; CEBPβ: CCAAT-enhancer-binding protein; YY-1: Yin Yang 1; BDNF: Brain-derived neurotrophic factor; CREB: cAMP response element binding protein; Nurr1: Nuclear receptor-related 1.
